# omu, a Metabolomics Count Data Analysis Tool for Intuitive Figures and Convenient Metadata Collection

**DOI:** 10.1128/MRA.00129-19

**Published:** 2019-04-11

**Authors:** Connor R. Tiffany, Andreas J. Bäumler

**Affiliations:** a Department of Medical Microbiology and Immunology, School of Medicine, University of California at Davis, Davis, California, USA; Indiana University, Bloomington

## Abstract

Metabolomics is a powerful tool for measuring the functional output of the microbiota. Currently, there are few established workflows for analysis downstream of metabolite identification. Here, we introduce omu, an R package designed for assigning compound hierarchies and linking compounds to corresponding enzyme and gene annotations for organisms of interest.

## Abstract (precis)

Metabolomics is a powerful tool for measuring the functional output of the microbiota. Currently, there are few established workflows for analysis downstream of metabolite identification.

## ANNOUNCEMENT

The omu R package is designed to analyze processed metabolomics count data. The central idea behind omu is assigning hierarchical metadata from the Kyoto Encyclopedia of Genes and Genomes (KEGG) (1) to each metabolite in order to help users create intuitive figures for visualizing their data. To do this, omu provides a suite of graphing and statistical functions centered around the use of assign_hierarchy, which provides the metadata for each metabolite based on a KEGG identifier in the user’s data. omu comes with an example data set of the fecal metabolome of nitric oxide synthase 2 (NOS2)-deficient C57BL/6J mice 3 days after mock treatment (gavage with sterile water) or oral gavage with a single dose of streptomycin (20 mg/animal). This data set was used to generate the figure in this paper.

**FIG 1:**
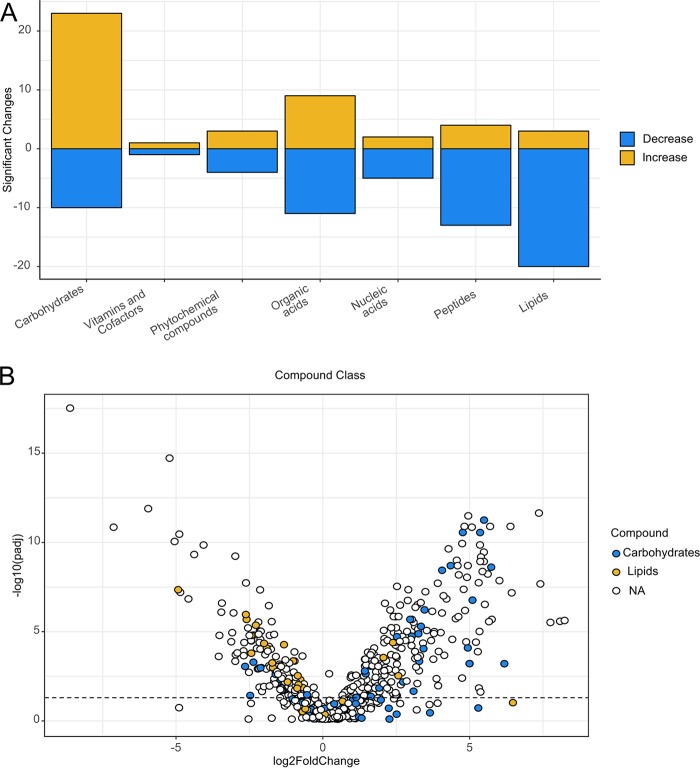
Data visualization in omu. (A) Bar plot showing the number of significantly different metabolites between treatment groups by class from the example data set that comes with the R package. (B) Volcano plot comparing two treatment groups from the example data set. Highlighted points in the plot correspond to metabolite classes.

Initially, users can use assign_hierarchy to provide metadata for each metabolite that has a KEGG compound number provided (Table 1). KEGG data are annotated by compound class, subclass 1, subclass 2, subclass 3, and subclass 4. This annotation provides several options for the users to analyze and visualize their data. For example, users can create a subset to a compound class they are particularly interested in, such as carbohydrates. The user can use the omu_summary function to perform a student’s *t* test on each metabolite between two experimental groups, provide a fold-change value for each compound, and use these data in conjunction with the hierarchical compound annotation to create intuitive figures. The count_fold_changes function can be used to provide a count table of every metabolite within a KEGG compound category that significantly increased or decreased between groups to make bar plots (Fig. 1A) that show how many compounds increased or decreased between experiment groups by a metabolite class or subclass. Alternatively, since omu_summary also provides fold-change data, the user can incorporate effect size into a figure by creating a volcano plot using the plot_volcano function, which allows the user to highlight points in the plot by the metadata (Fig. 1B).

**TABLE 1:** Data table created from the example data set, showcasing the metadata collection that omu can perform

Gene	Metabolite	KEGG no.	Metabolite count	Class	Subclass 1	Subclass 2	Species, strain, serotype
PA4091 (*hpaA*)	4-Hydroxyphenylacetic acid	C00642	32,307	Organic acids	None	None	Pseudomonas aeruginosa PAO1
N297_4221 (*hpaB*)	4-Hydroxyphenylacetic acid	C00642	32,307	Organic acids	None	None	P. aeruginosa PAO1, VE13
N296_4221 (*hpaB*)	4-Hydroxyphenylacetic acid	C00642	32,307	Organic acids	None	None	P. aeruginosa PAO1, VE2
PA14_11000 (*hpaA*)	4-Hydroxyphenylacetic acid	C00642	32,307	Organic acids	None	None	P. aeruginosa UCBPP, PA14
PSPA7_1007 (*hpaB*)	4-Hydroxyphenylacetic acid	C00642	32,307	Organic acids	None	None	P. aeruginosa PA7
PP4_31900 (*gbd*)	4-Hydroxybutyric acid	C00989	315	Organic acids	Carboxylic acids	Hydroxycarboxylic acids	Pseudomonas putida NBRC 14164
APT59_05510	4-Hydroxybutyric acid	C00989	315	Organic acids	Carboxylic acids	Hydroxycarboxylic acids	Pseudomonas oryzihabitans
PverR02_11545	4-Hydroxybutyric acid	C00989	315	Organic acids	Carboxylic acids	Hydroxycarboxylic acids	Pseudomonas veronii
BJP27_20245	4-Hydroxybutyric acid	C00989	315	Organic acids	Carboxylic acids	Hydroxycarboxylic acids	Pseudomonas psychrotolerans

The omu R package can also help users generate hypotheses from their metabolomics data. The KEGG_gather function can get all known enzyme orthology data and gene data associated with each metabolite from the KEGG database as long as the computer is connected to the Internet. This produces high-dimensional data, but assign_hierarchy also provides metadata for enzymes and for genes in the form of organism data, allowing the user to reduce the data to items of interest. For example, if a user is interested in genes that *Pseudomonas* spp. have to metabolize organic acids, the KEGG_gather function in conjunction with the assign_hierarchy function can be used to generate a table containing this information (Table 1).

In summary, omu is a novel metabolomics analysis tool that helps users describe their data by incorporating metabolite metadata into intuitive figures and creating tables with genes and enzymes associated with metabolites of interest.

### Data availability.

omu is available for download on the CRAN repository (https://cran.r-project.org/web/packages/omu/index.html) and was built using R (2), devtools (3), dplyr (4), ggfortify (5), ggplot2 (6), KEGGREST (7), knitr (8), magrittr (9), plyr (10), reshape2 (11), rmarkdown (12), stringr (13), roxygen2 (14), and tidyr (15). Detailed instructions for using omu can be found at https://cran.r-project.org/web/packages/omu/vignettes/Omu_vignette.html.
